# A targeted approach to vaccine hesitancy

**DOI:** 10.1093/oxfimm/iqad007

**Published:** 2023-10-28

**Authors:** Meredith Leston, Simon de Lusignan, F D Richard Hobbs

**Affiliations:** Nuffield Department of Primary Care Health Sciences, University of Oxford, Oxford OX2 6GG, UK; Nuffield Department of Primary Care Health Sciences, University of Oxford, Oxford OX2 6GG, UK; Nuffield Department of Primary Care Health Sciences, University of Oxford, Oxford OX2 6GG, UK

## Abstract

This short communication makes the case for targeted vaccine research when attempting to counter hesitancy, especially amongst vulnerable or rarefied patient groups. Far from disincentivizing vaccination, the freedom to research and publicize the limitations of these technologies for certain groups and personalizing dosing, pacing, adjuvants, and time-sensitive alternatives in response is essential for optimizing health outcomes while neutralizing the vaccine research landscape itself. Vaccine evangelism only arouses suspicion when it is not tempered by rigorous research into differential vaccine benefit-risk in this way. That said, the long-standing politicization of vaccination—a topic vulnerable to misinterpretation and media sensationalism—along with the commercial incentives associated with universal adoption makes more comparative and critical research difficult to fund and promote in practice. Likewise, a prescriptive approach to vaccination does little to address the issues of vaccine inequality that contribute to both hesitancy and conspiracy globally and will likely prove financially prohibitive in certain markets. These obstacles are not insurmountable, however, provided that comparative research is centrally subsidized, regulations ensure that vaccine development trials explore differentiated outcomes, especially amongst high-risk or rare groups, and findings are used to prioritize global vaccine allocation to those that stand to benefit most from them.

Even prior to the emergence of SARS-Cov-2, vaccine hesitancy appeared in the World Health Organisation’s 10 leading threats to global health [1]. Here, widespread refusal or reluctance to take up invitations to vaccinate were already reversing major steps forward against vaccine-preventable illness [2], most notably amongst the mumps, measles and rubella remit where herd immunity requirements for conferring protection are especially stringent [3]. Vaccine hesitancy had also proven itself to be contagious: scares affecting vaccination against one disease were seen to trigger declines in the uptake of those against others [4]. That said, for better or worse, there can be little doubt as to the catalytic role the pandemic has played for awareness-raising on this issue. 2021 marked the year the term ‘infodemic’ became common parlance [5] and that vaccine mandates or ‘liberation’ were promoted to political centrepieces [6]. While uptake of COVID-19 vaccines remained impressively high, especially amongst clinical risk groups, pockets of refusal or low uptake seen globally were still of concern [7].

However, it would be wrong to conceptualize such vaccine hesitancy as static or in any way singular with work by Kumar and colleagues [8] characterizing 6 discrete phases of hesitancy across the COVID pandemic alone (eagerness, ignorance, resistance, confidence, complacency and apathy). It is this heterogeneity that makes vaccine hesitancy so resistant to one-sized interventions [9]. While commentators are quick to attribute such trends and their consequences to misinformation and low levels of health literacy amongst the general public [10], the scientific community must do more to acknowledge its own contributions to varying forms of vaccine hesitancy—both historical and contemporary. For example, it must be acknowledged that much of the knowledge base underpinning vaccination, and its resultant portfolio of products, are built on the rolled-up sleeves of unwilling participants [11–14] and continue to underserve high-risk or marginalized groups [15].

These nuances within vaccine hesitancy are not reflected in the vaccine research landscape that aims to tackle it. There is therefore a real need to establish more specialized vaccine benefit-risk profiles than the mass market focus of the pharmaceutical complex currently allows for. For example, despite their well-documented vulnerability to vaccine-preventable illness [16], the immunosuppressed are still overwhelmingly excluded from the development trials that create the pharmacovigilance capable of countering hesitancy [17]. What little targeted research is available either collapses the immunosuppressed spectrum into one overfitting risk group [18] or narrows in on dominant subsets at the exclusion of all others [19]—potentially erasing subtrends of clinical relevance via either process. With ‘uncertainty about immune response given an underlying immunodeficiency’, ‘unknown long-term side effects of COVID-19 vaccination’, ‘pre-existing history of allergic reactions’, ‘limited amount of data’ and ‘lack of investigation of safety and effectiveness of COVID-19 vaccines in those with medical conditions’ serving as the most often-cited reasons that nearly 20% of a recent immunosuppressed cohort reported they were ‘somewhat’ or ‘very unlikely’, ‘unlikely’, or ‘not planning to get vaccinated’ [20], the value of addressing such knowledge gaps for the vaccine hesitancy agenda becomes obvious.

Furthermore, unlike any other product or procedure in medicine, we do not currently expect our doctors to know what brand, dose or schedule of vaccine would prove best for us despite having the both the data and maturity in clinical informatics to find out [21]. Likewise, time-sensitive vaccine alternatives, including monoclonal antibodies and next-generation antivirals, are prioritized for certain groups and withheld from others without much in the way of supporting literature [22]. By working to pinpoint those underserved by mainstream courses of vaccination, we can better prevent the instances of breakthrough infection and critical outcomes amongst the fully inoculated that undermine vaccine confidence [23] and make quick adjustments when these events do occur as opposed to dismissing them as medical inevitability. Such specialized research would not be motivated to disincentivize vaccination in any way, but would identify patients needing additional doses, medical supervision, and support during periods of high transmission. Even while running against immediate commercial objectives, this combination of radical transparency [24] and precision vaccinology will help to neutralize the vaccine research landscape while generating the evidence base needed for the more prescriptive approach that has been called for [25].

Ensuring the decision to vaccinate feels as personable and personalized as possible for patients is a vital first step for improving uptake [26] while countering prevailing accusations of paternalism in the vaccine space [27]. However, such a paradigm shift will not prove easy. As already alluded, it will be difficult to find those willing to fund research that might undermine the credibility of products launched after years, if not decades, of research and development; discovering instances of under-performance, non-response or elevated risk may be in the public interest, but it is not in the commercial interest of manufacturers. Moreover, actioning and disseminating findings of this nature should be handled with great care and couched in their proper context. For example, evidence of vaccine under-performance in specific risk-groups must inform policymaking, but not direct it. Said findings cannot be generalized to preclude patients from inoculation but can instead be used as important talking points between clinicians and their patients to improve the consultation experience and individual decision-making. Public trust in vaccines is also exceptionally delicate, as demonstrated by the misrepresentation and politicization of clotting risks associated with the Oxford-AstraZenca COVID vaccine [28] and the precautionary suspensions to its roll-out in Europe that resulted [29]. Likewise, the dangerous persistence of Wakefield’s linkage between MMR inoculation and autism [30] in the public imagination demonstrates the speed and longevity with which misleading claims can become sensationalized—especially when initially backed by the esteem of high-impact journals [31] or when amplified online by algorithms that prioritize user engagement over education [32]. Without intensive counter-messaging, such criticisms have been seen to anchor public discourse on vaccination rather than enhance it; this has only exacerbated vaccine hesitancy in certain populations [33].

Personalizing vaccine benefit-risk profiling will also depend upon the active championing of scientific investigators and academic outlets. However, long-standing issues of interpretation bias and publication bias [34] will have to be addressed before precise, dissenting, or null findings are given parity with population studies that return broadly positive results. This is especially true of evidence that only pertains to narrow interest-groups such as rarefied or complex patients. Having this research funded, published, and amplified is an uphill battle—even more so when findings run against corporate or national economic interests or a prevailing narrative of effectiveness amongst the general population. Such findings are highly inconvenient when there is mounting political pressure to re-open economic life, for example. Moreover, much of outlets’ reluctance in publishing and investigators’ aversion to pursuing these moderating views are embedded in the same identity politics both claim to counter [35]. As scientists, as supposed ‘intellectuals’, there is an unspoken pressure to take a universally positive, borderline evangelical, view of vaccination [22, 36]—something that can quickly amount to dogmatism if conflicting views or findings are not taken up with the same enthusiasm or are dismissed out of hand. Those whose work either modifies or counters this narrative can fall foul of misinformation algorithms or meet especially scathing peer review and government interference [37]. While it is important to contradict unfounded anti-vax sentiment, doing so with the same inflexibility and defensiveness is ill-advised. It merely perpetuates vaccine tribalism as accusations of elitism, bias or profiteering prevent rigorous evidence from allaying the concerns of the hesitant.

Finally, it is important to note the apolitical forms of vaccine hesitancy and obstacles that exist to achieving precision vaccinology. With access still far from equal [38], vaccine hesitancy is as much infrastructural as it is psychological [39]. Resource-constrained health systems and inequitable vaccine supply chains will never be able to meet the demands of personalized vaccine consultation and distribution [40]. This is an agenda that warrants global prioritization and centralized subsidization: improving the precision of vaccine benefit-risk profiling ensures that resources are prioritized appropriately and that those seen to be conferred comparatively less immunity through vaccination are signposted to the wraparound health services and products they need to survive infection. This information is of as much economic value to the NHS as it is to global vaccine distribution efforts such as GAVI, the Vaccine Alliance. Moreover, ensuring all nations’ highest-risk citizens are characterized and catered to by vaccine research will address the accusations of vaccine nationalism and western interference that have been seen to exacerbate hesitancy globally [41].

This short commentary has demonstrated the ways in which better targeting vaccine research and policies can counter the diverse underpinnings of hesitancy. The obstacles it has summarized can be addressed through a combination of subsidized research efforts—especially those that explore differentiated outcomes or ensure new products are tested to satisfaction within high-risk or rare patient groups—and the self-reflection of investigators and journals as to their own investigation and publication biases against moderating or dissenting views. Furthermore, although the COVID-19 pandemic has been seen to exacerbate ongoing issues of vaccine hesitancy, the global coordination of research efforts it engendered has proved the greatest asset for precision vaccinology [42]. Research such the OCTAVE Trial [43] into differential COVID vaccine response amongst the clinically vulnerable are the green shoots for this moon-shot, serving as the gold standard as to what can be achieved for underserved patients and their understanding of personal vaccine benefit-risk.

## Data Availability

This work does not offer any original data. As such, no data is available for request.
